# Synergistic Action of Antimicrobial Peptides and Antibiotics

**DOI:** 10.3390/ijms27104553

**Published:** 2026-05-19

**Authors:** Alicja Matyjewicz, Agata Krakowska, Dominik Műller, Jakub Krakowski, Beata Paczosa-Bator, Tomasz Skalski

**Affiliations:** 1 Department of Analytical Chemistry and Biochemistry, Faculty of Materials Science and Ceramics, AGH University of Krakow, Al. Mickiewicza 30, 30-059 Krakow, Poland; amatyjewicz@agh.edu.pl (A.M.); domul@agh.edu.pl (D.M.); paczosa@agh.edu.pl (B.P.-B.); 2 Department of Inorganic Chemistry and Pharmaceutical Analytics, Faculty of Pharmacy, Jagiellonian University Medical College, 9 Medyczna Street, 30-688 Kraków, Poland; 3 Department of Glass Technology and Amorphous Coatings, Faculty of Materials Science and Ceramics, AGH University of Krakow, Al. Mickiewicza 30, 30-059 Krakow, Poland; jkrakowski@agh.edu.pl; 4 Biotechnology Centre, Silesian University of Technology, Krzywoustego 8 Street, 44-100 Gliwice, Poland

**Keywords:** antimicrobial peptides, antibiotics, synergistic

## Abstract

In recent years, the rapid rise of antimicrobial resistance has intensified the search for alternative agents to treat drug-resistant infections. Antimicrobial peptides (AMPs) are promising therapeutic candidates due to their broad-spectrum activity, diverse mechanisms of action, relatively low risk of resistance development, and potential for use in combination therapies. This review outlines current knowledge on the properties and mechanisms of action of AMPs compared to conventional antibiotics. Furthermore, it discusses synergistic interactions between antimicrobial peptides and antibiotics, focusing on the underlying mechanisms, therapeutic implications, and translational challenges. It also summarizes key in vitro and in vivo studies, demonstrating enhanced antimicrobial efficacy of AMP–antibiotic combinations, including mechanisms such as increased membrane permeability, disruption of intracellular pathways, inhibition of biofilm formation, and efflux pump inhibition. The immunomodulatory and wound-healing properties of AMPs are also highlighted as factors that further strengthen their therapeutic potential in vivo. The review concludes with an overview of the main limitations hindering clinical translation and highlights ongoing research efforts aimed at optimizing AMP-based combination therapies against multidrug-resistant pathogens.

## 1. Introduction

The discovery of antibiotics remains one of the most important milestones in medical history, substantially reducing mortality rates and the spread of infectious diseases. The period between the 1930s and 1960s is often referred to as the “golden age” of antibiotic discovery [1]. Despite continuing advances in medicine and technology, there has been a pronounced reduction in the discovery and market introduction of novel antibiotics. This poses a major challenge due to increasing antimicrobial resistance (AMR) [2,3].

Antimicrobial resistance arises through several well-characterized mechanisms that enable bacteria to survive antibiotic exposure. These include: reduced antibiotic uptake through the membrane (e.g., via the decreased number of bacterial porins), active efflux systems that expel antimicrobial compounds, modification of antibiotic targets (such as ribosomal subunits, penicillin-binding proteins, DNA gyrase and topoisomerase enzymes, or peptidoglycan precursors), enzymatic inactivation of antibiotics, and the spontaneous mutations in bacterial DNA [4,5]. Importantly, resistance determinants can spread between species and environments through horizontal gene transfer, including conjugation, transformation, transduction, and vesicle-mediated transfer [5].

Today, antibiotics are extensively used not only in human medicine but also in food production and animal husbandry, which has led to a continuous increase in global antibiotic consumption [6]. However, this widespread use—particularly when antibiotics are misused or overprescribed—has greatly contributed to the growing problem of antimicrobial resistance [7]. Over the past decades, there has been a dramatic rise in the number of human pathogens resistant to one or more antibiotics worldwide [8]. Prolonged antibiotic exposure promotes the development of resistant strains, often resulting in cross-resistance within the same antibiotic class [9]. A major challenge in hospital-acquired infections is posed by a group of multidrug-resistant (MDR) pathogens collectively referred to as ‘ESKAPE’. This acronym encompasses six clinically important taxa that are especially difficult to treat due to their resistance profiles: *Enterococcus faecium*, *Staphylococcus aureus*, *Klebsiella pneumoniae*, *Acinetobacter baumannii*, *Pseudomonas aeruginosa*, and *Enterobacter* spp. [1]. It is estimated that infections caused by drug-resistant microbes are currently responsible for at least 700,000 deaths annually. According to projections by the World Health Organization, in the worst-case scenario this number could rise to 10 million annually by 2050 if effective preventive and therapeutic measures are not implemented [10].

Therefore, the increase in antibiotic resistance has become a global scientific challenge. New strategies that enhance the efficacy of existing antibiotics and prevent the development of resistance are crucial to address this growing crisis in the future. Among the more promising strategies is the use of synergistic therapies, especially those that combine different antibiotics to more effectively treat drug-resistant infections [11].

While recent literature has increasingly recognized the potential of AMP–antibiotic combinations, many reviews focus heavily on specific pathogen classifications or bacterial adaptive resistance mechanisms. To distinguish this review and provide a structured analytical perspective, we introduce a comprehensive conceptual framework classifying the synergistic mechanisms into four primary categories: (I) membrane permeabilization, (II) biofilm inhibition and disruption, (III) direct potentiation of intracellular activity, and (IV) inhibition of resistance mechanisms. Furthermore, this review critically addresses the often-overlooked translational gap by analyzing the methodological flaws in current in vitro synergy testing (such as assay discrepancies and media variations) and contextualizing them against real-world clinical trial outcomes of peptide-based therapeutics. This dual focus on mechanistic categorization and translational bottlenecks serves as a foundational guide for designing future combination therapies. In recent years, antimicrobial peptides (AMPs) have emerged as potential novel therapeutics against bacterial resistance, particularly since their early clinical applications have revealed broad-spectrum efficacy. Interestingly, the antimicrobial properties of peptide-rich larval secretions were already exploited in early maggot-based wound therapy described by Baer in 1930, although the peptide nature of these antimicrobial factors was only recognized decades later [12,13]. As key effectors of innate immunity, AMPs are the cornerstone of the host’s first-line defense. They exert multifaceted antimicrobial actions, including direct lysis of pathogens, inhibition of transcription and translation at the ribosomal level, and modulation of immune responses through chemoattraction of leukocytes [14,15] and enhancement of cytokine production [16]. The primary mechanism involves disruption of phospholipid membrane permeability via cationic interactions with negatively charged phospholipids in bacterial membranes—a predominantly physical process that minimizes adaptive resistance in target microorganisms. This multi-target activity, which limits the impact of bacterial mutations that typically drive resistance, coupled with a low propensity for resistance development, positions AMPs as promising candidates for next-generation antimicrobial therapies [17,18].

Antimicrobial peptides are a diverse group of small, naturally occurring molecules, typically composed of 12 to 50 amino acid residues. They are found in a wide range of species, including humans, plants, insects, and microorganisms, and play key roles in innate immunity [19]. In addition to naturally occurring antimicrobial peptides, the design of synthetic AMPs is rapidly advancing, with such peptides most often optimized to increase antimicrobial activity, stability, and reduce toxicity [20,21]. The majority of AMPs are cationic and amphipathic in nature. These properties allow them to associate with negatively charged microbial membranes, causing membrane disruption. Structurally, they can adopt various conformations—such as α-helices, β-sheets, or extended structures—often dependent on environmental conditions and their amino acid sequence [22].

Functionally, AMPs act as rapid and wide-spectrum antimicrobial agents with activity against a broad range of pathogens, including Gram-positive and Gram-negative bacteria, fungi, viruses, and protozoa [23]. Their mechanisms of action are complex. In addition to direct membrane disruption, antimicrobial peptides can interact with intracellular targets such as nucleic acids, or interfere with essential physiological processes, including protein synthesis and enzyme activity [24,25]. Some AMPs are capable of neutralizing bacterial toxins, inhibiting pro-inflammatory responses and biofilm formation, as well as promoting wound healing. They may also modulate immune responses or exhibit synergistic effects with conventional antibiotics [11,26,27]. This broad activity profile and reduced tendency to induce resistance make AMPs a promising alternative or complement to traditional antibiotics, particularly in the face of rising antimicrobial resistance [11,28].

Cryptic antimicrobial peptides represent an underexplored class of AMPs with significant therapeutic potential. These peptides, encrypted within larger, functionally unrelated host proteins, differ significantly in their amino acid composition and present an underexplored reservoir for future synergistic therapies [29,30]. Cryptic AMPs differ in their amino acid composition, having a higher proportion of hydrophobic and basic amino acids compared to classical AMPs. Furthermore, their mechanism of action is primarily related to damaging and increasing the permeability of the outer bacterial cell membrane, leading to cell death. Some cryptic AMPs can also act synergistically or additively when combined, further emphasizing their potential as a source of future antimicrobial agents [30].

The growing threat of antimicrobial resistance makes it essential to explore new treatment strategies that could help restore the effectiveness of existing antibiotics. A particularly promising approach is the use of antimicrobial peptides in combination with conventional antibiotics. Such combinations can improve antimicrobial effectiveness through synergistic or additive interactions while also helping to lower the doses needed and reduce potential toxicity [22,31]. In this context, synergy refers to an interaction between two agents in which the combined effect exceeds the sum of their individual effects, whereas additivity describes a situation where the combined effect corresponds to the sum of their separate activities [32]. By disrupting microbial membranes, AMPs facilitate the entry of antibiotics into bacterial cells, where they can effectively target essential intracellular processes such as DNA replication, protein synthesis, and cell wall formation. This mechanism is key to their demonstrated synergistic potential, especially against biofilm-forming and highly resistant pathogens [22]. However, the effectiveness of combination therapy depends on several factors. These include the choice of compounds, dosing schedules, specific pathogens involved, resistance mechanisms, and the conditions of the infection site. A comprehensive understanding of these factors, along with a clear explanation of the mechanisms underlying their synergy, is crucial for optimizing treatment protocols. As research advances, combinations of AMPs and antibiotics have the potential to become a fundamental part of next-generation antimicrobial therapies, providing a sustainable approach to addressing the global challenge of antibiotic resistance [11,33].

The complementary mechanisms of action between antimicrobial peptides and traditional antibiotics frequently produce synergistic effects that improve overall antimicrobial efficacy. To systematically understand and apply these interactions in clinical design, we propose a classification framework for AMP–antibiotic synergy based on four distinct mechanistic pillars:

- Mechanism I: Increased Membrane Permeability: AMPs cause the breakdown of bacterial membranes by increasing permeability or causing structural defects, allowing antibiotics that might otherwise have difficulty reaching their targets to enter cells. This disruption compromises membrane integrity through mechanisms such as the carpet model, toroidal pore formation, or barrel-stave insertion.
- Mechanism II: Biofilm Inhibition and Disruption: AMPs exert their action on biofilms by preventing initial bacterial cell adhesion, degrading biofilm matrix components, and downregulating the expression of genes responsible for biofilm formation. This facilitates antibiotic access by promoting biofilm dispersal.
- Mechanism III: Direct Potentiation of Antibiotic Activity: Beyond membrane disruption, AMPs can penetrate bacterial cells to disrupt intracellular metabolic processes and interfere with the synthesis of essential components, such as DNA replication, RNA transcription, and protein synthesis.
- Mechanism IV: Inhibition of Resistance Mechanisms: AMPs can restore or enhance the activity of conventional antibiotics against multidrug-resistant bacteria by interfering with resistance mechanisms. For example, AMPs can directly block the action of efflux pumps or disrupt the proton-motive force that drives them, thereby enhancing intracellular antibiotic accumulation.

Innovative strategies based on a deeper understanding of existing antimicrobial agents are essential to combat the growing threat of antimicrobial resistance. This review outlines the individual mechanisms of action of both antimicrobial peptides and conventional antibiotics, and describes the basis for their synergistic interactions. It also highlights key experimental findings and current challenges in applying these synergistic approaches to clinical practice.

## 2. Antimicrobial Peptides and Antibiotics: Individual Modes of Action

Before detailing their distinct modes of action, it is important to acknowledge that the boundary between AMPs and ‘conventional antibiotics’ can sometimes appear artificial. Many traditional antibiotics are chemically peptides themselves, such as glycopeptides (e.g., vancomycin) or streptogramins. For the scope of this review, we define ‘AMPs’ primarily as host defense peptides—originating from the innate immune systems of multicellular organisms or structurally similar synthetic analogs—that predominantly rely on rapid, physical disruption of lipid membranes. In contrast, ‘conventional peptide antibiotics’ are typically microbial secondary metabolites that target highly specific bacterial receptors or enzymes, such as the binding of glycopeptides to the D-alanyl-D-alanine precursors to inhibit cell wall synthesis. However, there are agents that bridge these categories, such as polymyxins. While clinically classified and utilized as conventional antibiotics, polymyxins are microbial lipopeptides whose membranolytic mechanism of action closely mirrors that of cationic AMPs. To maintain consistency, agents like polymyxin will be referred to herein as peptide antibiotics, while acknowledging their shared mechanistic traits with AMPs. The effectiveness of AMPs comes from their ability to use diverse mechanisms of action, making them powerful and versatile agents in the fight against microbial infections. AMPs can directly disrupt bacterial membranes, modulate the immune response, regulate inflammation, and interfere with intracellular targets [24,34]. Their activity depends on multiple factors, including their size, amino acid sequence, three-dimensional conformation, net charge, hydrophobicity, and amphipathic character [35].

Based on their mechanism of action, antimicrobial peptides are divided into those that act on the membrane and those that do not act on the membrane [36]. AMPs access their bacterial targets through two distinctly different primary routes depending on their structural classification: direct membrane disruption or non-destructive transporter-mediated penetration. The majority of characterized AMPs are membranolytic, ultimately causing cell lysis. In this pathway, initial interaction is driven by electrostatic and hydrophobic interactions between the positively charged L-arginine and/or L-lysine residues of the AMPs and the negatively charged regions of bacterial membranes (e.g., teichoic acids in Gram-positive bacteria and lipopolysaccharides in Gram-negative bacteria) [22,26,37]. Conversely, a separate class of non-membranolytic AMPs—such as proline-arginine-rich antimicrobial peptides (PrAMPs)—penetrates the bacterial cell envelope without compromising membrane integrity, permeability, or potential. Instead of damaging the lipid bilayer, these peptides hijack specific inner membrane transporter proteins, such as SbmA or MdtM, to actively translocate into the cytoplasm. Once inside, they bind to intracellular targets, such as the ribosome, to block the process of translation [38,39]. Recognizing this distinction is essential when evaluating how different AMPs synergize with conventional antibiotics. Among membrane-active AMPs, several mechanistic models have been proposed to explain how these peptides compromise membrane integrity.

In the barrel-stave model, the antimicrobial peptides insert into the lipid bilayer of the bacterial cell membrane and lead to the formation of transmembrane channels or pores with inward-facing hydrophilic fragments. The formation of pores affects membrane functionality, disrupting the transmembrane potential and ion gradients. This results in increased membrane permeability, uncontrolled flow of ions and molecules, and ultimately, bacterial cell death [23,26].

The second mechanism is the toroidal pore model, in which AMPs aggregate on the surface of the bacterial cell membrane, inducing the inward bending of the lipid monolayer and pore formation. In this model, the hydrophilic regions of peptides are inserted into the membrane and anchored to the polar head groups of the phospholipids. The pores are lined by both the peptides and the lipid head groups, ultimately compromising membrane integrity and leading to cell death [26,37,40].

The third mechanism is the carpet model, in which AMPs are positioned parallel to the surface of the bacterial membrane and bind electrostatically to negatively charged lipid components. This surface coverage is similar to a “carpet” that interferes with lipid packing and increases the membrane permeability. At a critical concentration, the peptides destabilize the membrane integrity, causing disintegration into tiny fragments or micelles and ultimately cell lysis [23,37,41].

**Figure 1:**
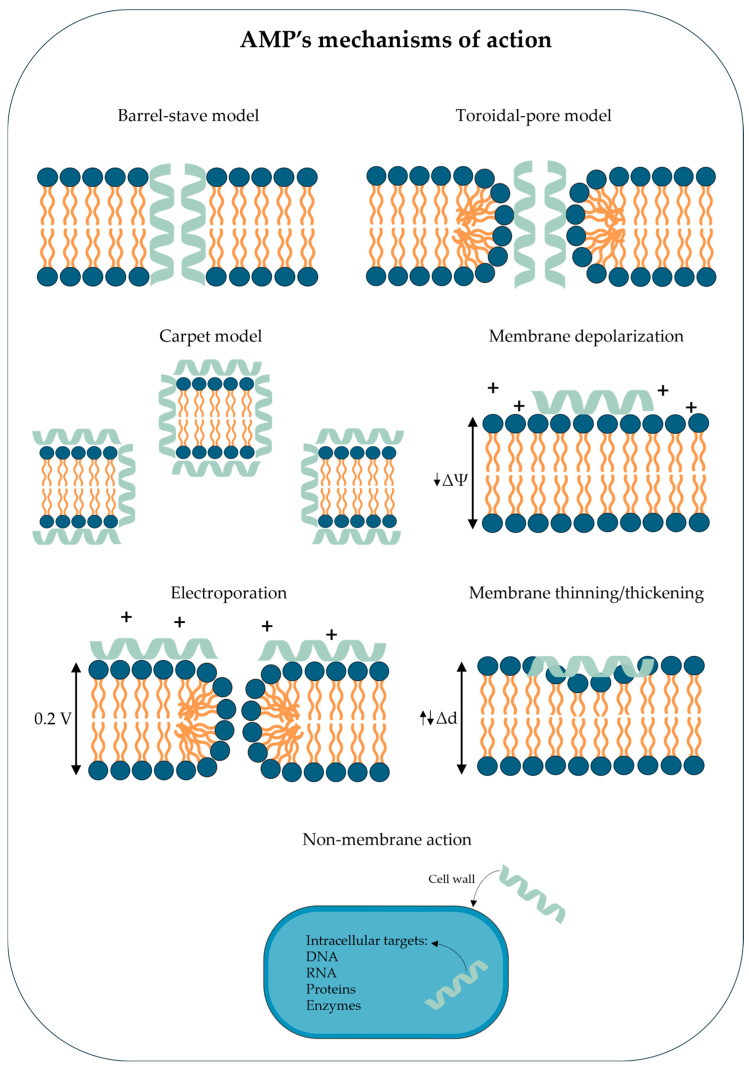
Mechanism of action of antimicrobial peptides (AMPs).

In addition to these three classical models, other mechanisms of membrane perturbation have also been reported. Some AMPs can increase the electrostatic potential to at least 0.2 V on both sides of the bilayer upon accumulation on the membrane surface, leading to molecular electroporation and pore formation [42,43]. Another mechanism of action involves membrane depolarization through the disruption of the ion gradient, leading to a loss of membrane potential [18,44]. Antimicrobial peptides can also disrupt membranes through non-porous mechanisms such as membrane thinning, where shallow peptide insertion reduces bilayer thickness and promotes local destabilization, or membrane thickening, where deeper insertion increases lipid packing and creates hydrophobic mismatch that may affect membrane organization [45]. All of these models ultimately compromise membrane integrity, leading to cellular lysis. However, it is crucial to distinguish membranolytic AMPs from a separate class of non-membranolytic peptides that exert their primary activity entirely inside the cell. Generally, AMPs that interact with intracellular targets do not affect membrane integrity, permeability, or membrane potential. Instead of damaging the lipid bilayer, these peptides penetrate the bacterial cell envelope by exploiting specific transport proteins. For example, proline- and arginine-rich peptides frequently utilize the SbmA transporter to translocate across the inner membrane without causing disruption. Once they accumulate in the cytoplasm, they interact with intracellular molecular components, leading to the inhibition of nucleic acid (DNA and RNA) and protein synthesis (e.g., by directly binding to ribosomes and blocking translation), disruption of enzymatic functions, or interference with protein folding [38,39,46]. The ability of AMPs to act on multiple intracellular pathways demonstrates their potential therapeutic applications for multidrug-resistant pathogens [24,37,41,47]. The diverse mechanisms of action of the antimicrobial peptides described above are summarized schematically in Figure 1.

While antimicrobial peptides characteristically rely on rapid, physical membrane disruption, the vast majority of conventional antibiotics target specific intracellular processes or cell wall synthesis. While the list of available antibiotics is vast, their most prominent classical mechanisms include the inhibition of bacterial cell wall biosynthesis, the inhibition of nucleic acid and/or protein synthesis, and the disturbance of essential metabolic pathways [48]. Although a select minority of conventional agents (such as the aforementioned lipopeptides and peptide antibiotics) also target cell membrane integrity, they represent an exception within the broader classical antibiotic arsenal.

Cell wall-targeting antibiotics are one of the most potent and most used antibacterial agents [49]. The cell wall, composed primarily of peptidoglycan, is essential for bacterial viability, growth, and shape maintenance [50]. Two major classes of antibiotics whose modes of action lead to inhibition of cell wall synthesis are the β-lactams and the glycopeptide antibiotics [1]. β-lactam antibiotics bind to penicillin-binding proteins (PBPs), leading to disruption of the final stage of peptoglycan synthesis, thereby weakening the cell wall and ultimately leading to cell death [1,51]. Glycopeptides, on the other hand, bind to the D-alanyl-D-alanine portion of the side chain of the peptide subunit of the peptidoglycan precursor. As a result, they prevent the subunit from binding to PBPs, thereby inhibiting cell wall synthesis [52].

Another crucial category—and arguably the largest class of antibiotics used in clinical practice—comprises agents that inhibit bacterial protein synthesis. Indeed, as highlighted by Sohmen et al. (2009) [53], the majority of clinically relevant antibiotics target the bacterial ribosome, which is composed of two subunits: the small 30S and the large 50S subunits [54]. Due to the functional complexity of the ribosome, antibiotics interfere with multiple stages of translation. They may block tRNA binding, inhibit mRNA translocation, or prevent peptide bond formation [55]. Classes of antibiotics that target bacterial protein synthesis include aminoglycosides, macrolides, tetracyclines, streptogramins, phenicols (e.g., chloramphenicol), and lincosamides (e.g., clindamycin) [51]. Aminoglycoside bind to the 30S ribosomal subunit, causing misreading of mRNA code and incorporation of incorrect amino acids into growing peptide chains, resulting in premature termination of mRNA translation [1,52]. Tetracyclines also act on the 30S subunit, but instead block the attachment of aminoacyl-tRNA to the ribosomal A-site, preventing elongation of the peptide chain [51]. In contrast, macrolides, lincosamides, and streptogramin B antibiotics bind to the 50S subunit, where they interfere with ribosomal translocation and may cause early detachment of incomplete peptides [1,52].

Another class of conventional antibiotics targets bacterial DNA, interfering with essential processes such as DNA replication, repair, or transcription. DNA damage induced by antimicrobial agents can occur through several mechanisms, including direct chemical damage, interference with DNA-associated proteins, and secondary effects of metabolic disruption [56]. Antibiotics that directly affect DNA replication include fluoroquinolones [57]. In contrast, sulfonamides do not act directly on DNA but inhibit folate metabolism, thereby limiting the availability of cofactors required for nucleotide biosynthesis. Fluoroquinolones inhibit DNA synthesis by targeting DNA gyrase in Gram-negative bacteria and topoisomerase IV in Gram-positive bacteria [58]. Other antibiotics, such as rifampicin, interfere with bacterial RNA polymerase, effectively inhibiting transcription initiation. Although rifampicin does not directly damage DNA, its interference with gene expression at the nucleic acid level contributes to its bactericidal activity [59,60].

Antibiotics can also interfere with important bacterial metabolic pathways. One of the best-known examples is the inhibition of folic acid metabolism by sulfonamide and trimethoprim antibiotics, which are often used in combination to achieve a synergistic effect [58]. Trimethoprim binds to the active site of the dihydrofolate reductase (DHFR), disrupting thymidylate synthesis and DNA replication. Sulfonamides, on the other hand, act as structural analogs of para-aminobenzoic acid (PABA), a substrate required for dihydropteroate synthase (DHPS) activity. This competition leads to the inhibition of folic acid production and, consequently, the blocking of the synthesis of purines, pyrimidines, and certain amino acids essential for DNA and protein production [1,51].

In addition to their direct effects on bacterial metabolic pathways, antibiotics can increase metabolic stress in the cells. Antibiotic-induced damage to high-energy processes such as protein biosynthesis and DNA replication further disrupts the metabolic homeostasis of bacterial cell. These primary damages trigger stress response pathways that involve increased metabolic activity to mitigate cellular damage [61]. However, increased metabolic flux is also associated with an increase in reactive metabolic byproducts (RBPs), including reactive oxygen species (ROS), which contribute to the lethal effects of antibiotics [62]. Increased production of harmful hydroxyl radicals can disrupt central metabolism pathways, such as the tricarboxylic acid (TCA) cycle and iron metabolism, ultimately leading to cell death [60]. Understanding the downstream effects of conventional antibiotics is essential for designing more effective combination therapies.

Despite their initial effectiveness, monotherapies using conventional antibiotics are increasingly limited by the rapid evolution of antimicrobial resistance (AMR) [34]. In response to selective pressure from their environment, bacteria can develop resistance mechanisms, which compromise the effectiveness of the antibiotic therapy. The growing global use of antibiotics—particularly in developing countries—accelerates the emergence of AMR, leading to increased disease and mortality rates [63]. Bacteria become resistant as a result of horizontal gene transfer and mutations, which leads to ineffective therapy [64]. Moreover, monotherapy often fails to eliminate all bacterial cells, leaving behind persisters or tolerant variants that can reignite infection [65]. These limitations highlight the urgent need for alternative approaches, including combination therapies, new antimicrobial agents, and host-targeted strategies that minimize resistance development. Antimicrobial peptides have emerged as promising candidates, either as standalone agents or in synergy with traditional antibiotics, due to their rapid and multi-step modes of action particularly membrane perturbation [34].

## 3. Mechanisms Underlying Synergistic Interactions

The complementary mechanisms of action between antimicrobial peptides and traditional antibiotics frequently produce synergistic effects that improve overall antimicrobial efficacy [66]. AMPs often cause the breakdown of bacterial membranes by increasing permeability or causing structural defects, allowing antibiotics that might otherwise have difficulty reaching their targets the enter cells [11]. This combined strategy can minimize concentration-dependent toxicity or adverse effects, increase bacterial killing, and lower the required antibiotics doses [27]. Additionally, because the cell membrane has a less defined structure and may be less prone to the development of resistance, agents that target it may be preferred [67]. As previously described, antimicrobial peptides can disrupt bacterial membranes through mechanisms such as the carpet model, toroidal pore formation, or barrel-stave insertion. These disruptions compromise membrane integrity and lead to increased permeability [23].

This increase in membrane permeability can be used to potentiate antibiotics that typically face difficulty penetrating bacterial envelopes, offering a basis for synergistic therapeutic strategies. AMPs are a vast and diverse group of molecules that are part of the innate immune system, constituting the body’s first line of defense and cooperating with immune cells to eliminate pathogens and eradicate infections. In addition to antimicrobial activity, antimicrobial peptides also mediate immunomodulatory effects, chemotaxis, apoptosis, and wound healing [27,68,69]. Moreover, AMPs can be effectively chemically synthesized and strategically modified to improve their pharmacological properties and efficacy, adjust their spectrum of action, and reduce their toxicity and in vivo degradation [64]. Such structural optimizations—which include strategies like amino acid substitution (e.g., D-enantiomers), cyclization, PEGylation, and lipidation—collectively contribute to their adaptability and may help limit the development of resistance [70].

One easily modified antimicrobial peptide is anoplin [71]. Anoplin (GLLKRIKTLL-NH_2_) is a short, 10-amino acid peptide isolated from the venom of the wasp *Anoplius samariensis*. Its advantage is strong and broad antimicrobial activity against Gram-positive and Gram-negative bacteria, while maintaining low toxicity to mammalian cells and low hemolytic activity [72,73]. Its mechanism of action is based on the formation of an amphiphilic α-helical structure, interaction with anionic biological membranes and the formation of pores in them, which leads to membrane disruption [71,74]. One of the beneficial modifications of AMP is the addition of a β-amino acid, which provides secondary structure selection and protease resistance [75]. Both anoplin and two of its β-Ala modified analogues demonstrated synergistic effects in combination with antibiotics against *P. aeruginosa* ATCC 27853. The Ano-1β analogue (A_β_LLKRIKTLL-NH_2_) demonstrated synergy with rifampicin and polymyxin B, while Ano-8β (GLLKRIKA_β_LL-NH_2_) demonstrated synergy only with rifampicin. Additionally, they demonstrated the highest antimicrobial activity against all tested *P. aeruginosa* strains and were more stable, retaining antimicrobial activity in various pH environments [76].

Another promising example of AMPs use is the human cathelicidin LL-37, which exhibits antimicrobial activity by disrupting bacterial cell membranes, leading to cell lysis, against numerous Gram-negative and Gram-positive bacteria, including pathogens from the genera *Escherichia*, *Pseudomonas*, *Staphylococcus*, and *Enterococcus*. Although LL-37 has not yet been approved as a therapeutic agent, it is widely investigated as a potential antibacterial agent [77]. Combining LL-37 with bactericidal antibiotics such as amikacin has been shown to enhance antibacterial activity against *S. aureus*. This synergy is likely due to LL-37-induced membrane permeabilization, which facilitates antibiotic entry into bacterial cells. In contrast, for antibiotics acting extracellularly on the cell wall, such as amoxicillin (in combination with clavulanic acid), the mechanism of interaction with LL-37 may differ and is not solely attributable to enhanced intracellular uptake. No synergistic effect was observed with bacteriostatic antibiotics such as tetracycline or erythromycin, suggesting that the interaction depends on the antibiotic mode of action [78]. Furthermore, the combination of LL-37 with several antibiotics increased their effectiveness against *Clostridioides difficile*. LL-37 demonstrated synergistic effects with antibiotics such as meropenem and moxifloxacin, including against strains resistant to these agents. In 16 out of 20 tested strains, combining LL-37 with antibiotics significantly increased membrane depolarization, suggesting enhanced bacterial killing through compromised membrane integrity [79]. Additionally, LL-37 has been shown to exhibit anti-biofilm activity [80].

Biofilms are defined as fixed communities of microorganisms immersed in extracellular polymeric substance (EPS), composed of extracellular polysaccharides, DNA, and proteins [81,82]. Biofilm allows the microorganisms living within it to survive in harsh environmental conditions and protects them from the host immune system by impairing phagocyte and complement systems. This structure is believed to be the primary cause of nosocomial infections in immunocompromised individuals and increases resistance to conventional antibiotics by up to 1000 times [83]. The increased resistance of bacteria in biofilms compared to planktonic cells results from, among other factors, different morphology, physiology, and gene transfer. This particularly applies to antibiotics that affect cell division, such as β-lactams antibiotics. In biofilms, the availability of oxygen and nutrients is limited, which slows down the metabolism and the rate of growth and division [81]. Due to the antibiotic resistance of biofilm-forming microorganisms, alternative antimicrobial agents are necessary [84]. Furthermore, combination therapies are more beneficial than antibiotic monotherapies for combating biofilm formation [83].

AMPs exert their action on biofilms through several mechanisms, including inhibiting biofilm formation by preventing initial bacterial cell adhesion, disrupting biofilm structure by degrading biofilm matrix components, disrupting the membrane potential of biofilm-embedded cells, disrupting bacterial cell signaling, and downregulating the expression of genes responsible for biofilm formation and transport binding proteins [84,85,86]. Importantly, antimicrobial peptides have demonstrated synergistic effects with antibiotics in the treatment of biofilm-associated infections, facilitating antibiotic access by increasing bacterial membrane permeability and promoting biofilm dispersal [86]. In addition to its membrane-disrupting activity, LL-37 has been shown to interfere with biofilm formation and maintenance, making it a valuable candidate for combination therapies targeting biofilm-associated infections. Numerous studies [84,85] have shown that LL-37 targets many key biofilm formation processes, including limiting cell adhesion in the initial stage of biofilm formation, stimulating twitching motility, causing bacteria to wander across the biofilm surface, downregulating the expression of biofilm-associated genes, degrading the biofilm matrix. Additionally, LL-37 can deeply penetrate biofilm structure, where it reduces cell viability by creating pores in the cell membranes of embedded bacteria [80]. Building on these properties, LL-37 has been investigated in combination therapies to enhance antibiotic efficacy against biofilms. Ridyard et al. (2023) [87] demonstrated that the combination of LL-37 with the antibiotic polymyxin B (PMB) exhibited potent synergistic activity against *E. coli* MG1655 and *P. aeruginosa* PAO1 cells, both in planktonic form and in biofilms. A synergistic effect was also demonstrated for six resistant *E. coli* strains. Furthermore, combined treatment with PMB + LL-37 demonstrated better biofilm inhibition and eradication properties than individual treatments, which provides a basis for further research on the synergy between AMP and conventional antibiotics. These findings highlight the potential of AMP–antibiotic combinations as innovative approaches for overcoming multidrug resistance and treating persistent biofilm-associated infections.

## 4. Direct Potentiation of Antibiotic Activity

In most cases, the synergy between antibiotics and antimicrobial peptides is attributed to the ability of AMPs to increase bacterial cell membrane permeability, thereby enhancing the accessibility of antibiotics to intracellular targets. However, AMPs also have additional potential for synergistic interactions with antimicrobial agents [88]. They can enhance antibiotic efficacy not only through membrane disruption but also by affecting bacterial metabolism and stress pathways. As highlighted earlier, a distinct class of non-membranolytic AMPs can translocate into bacterial cells without compromising membrane integrity, typically by hijacking bacterial transport proteins. Once inside, they act synergistically with conventional antibiotics by disrupting intracellular metabolic processes and interfering with the synthesis of essential components. These coordinated actions include interfering with DNA replication, RNA transcription, protein synthesis, and disrupting the cellular energy supply [86,89,90]. Indolicidin, a member of the cathelicidin family, represents an AMP with multiple intracellular mechanisms of action. Although it exhibits modest membrane permeabilization and interacts with bacterial lipopolysaccharides to facilitate uptake, its primary activity is linked to interference with essential intracellular processes. Indolicidin penetrates the bacterial cell and binds tightly to bacterial nucleic acids, directly interfering with DNA synthesis and inhibiting essential process such as transcription. Moreover, this peptide also interacts with ATP and inhibits ATP-dependent enzymes, further crippling bacterial metabolism [89,91,92]. These non-membranolytic activities contribute to its ability to act synergistically with conventional antibiotics. In studies, indolicidin and its optimized variants demonstrated synergistic effects with several antibiotics, including polymyxin B, tobramycin, gentamicin and amikacin [92]. AMPs can also target RNA and protein biosynthesis, interfering with essential processes that ultimately lead to bacterial cell death [25]. The mechanism of action of pleurocidin involves a combination of membrane permeability and metabolic inhibition. In addition to membrane disruption, pleurocidin inhibits metabolic processes like DNA synthesis and migration and RNA synthesis, thereby inhibiting bacterial cells from protein synthesis [93]. Moreover, this peptide has demonstrated synergistic activity with antibiotics such as ampicillin, chloramphenicol, and erythromycin against both Gram-positive and Gram-negative bacteria [94]. Some AMPs exert their effects by inducing oxidative or metabolic stress. Li et al. (2017) [95] in their studies showed that the CLP-19 peptide not only has direct antibacterial activity but also shows synergy with bactericidal and bacteriostatic antibiotics. Its mechanism of action is based on the generation of hydroxyl radicals by transiently increasing the production of NAD+ (or depleting NADH), ultimately leading to oxidative stress and the lysis of bacterial cells. When combined with ampicillin, ceftazidime, or levofloxacin, CLP-19 demonstrated synergy, promoting the production of hydroxyl radicals against *E. coli* (ATCC 25922) and *S. aureus* (ATCC 29213).

**Figure 2:**
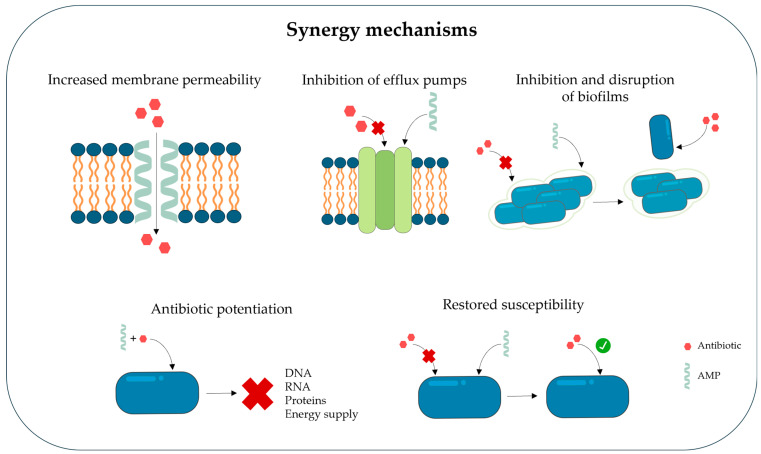
Mechanisms of synergy between antimicrobial peptides (AMPs) and antibiotics.

Together, these examples illustrate that AMPs can act synergistically with conventional antibiotics through multiple mechanisms. This multifaceted activity underlines their potential as versatile adjuncts in combination therapies aimed at overcoming antimicrobial resistance and treating persistent infections (Figure 2).

## 5. Inhibition of Resistance Mechanisms

Another important aspect of synergistic interactions between AMPs and antibiotics is their ability to interfere with bacterial resistance mechanisms. By targeting efflux pumps or limiting the emergence of resistance during treatment, AMPs can restore or enhance the activity of conventional antibiotics against multidrug-resistant bacteria.

One well-established resistance mechanism is the active transport of antimicrobial agents out of the cell using efflux pumps, which maintains the concentration of substances in the periplasm at a level that is non-toxic to the bacterial cell [89,96]. AMPs can act in two ways: by increasing cellular permeability or by directly blocking the action of efflux pumps, thereby enhancing intracellular antibiotic accumulation [89,97].

AMPs can interfere with efflux systems through two distinct mechanisms: altering protein expression and disrupting pump function. At the regulatory level, certain AMPs can downregulate the genetic expression of efflux pumps, reducing their abundance in the bacterial membrane. Alternatively, at the energetic level, many bacterial efflux pumps rely on the proton-motive force to actively expel drugs. Membrane-active cationic AMPs can collapse this transmembrane electrochemical gradient. By destroying the pump’s energy source, the AMP causes its immediate shutdown, trapping the antibiotic inside the cell and potentiating its intracellular activity [90]. For instance, Goldberg et al. (2013) [98] demonstrated that the C12(ω7)K-β12 peptide acts synergistically with tetracycline and erythromycin against *E. coli*, disrupting the function of efflux pumps by acting on the proton driving force and increasing antibiotic efficacy.

AMPs can also act on the bacterial cell walls to facilitate antibiotic uptake. Plectasin, a defensin derived from *Pseudoplectania nigrella*, exhibits potent bactericidal activity against Gram-positive bacteria. Its mechanism of action involves binding to the pyrophosphate moiety of lipid II in the bacterial cell wall, leading to rapid killing of the target bacteria. Its improved variant NZ2114 showed synergistic activity with the antibiotics amoxicillin, penicillin, flucloxacillin, gentamicin, neomycin or amikacin against methicillin-susceptible *Staphylococcus aureus* (MSSA) and methicillin-resistant *Staphylococcus aureus* (MRSA) [97].

In addition, some AMPs can restore the susceptibility of antibiotic-resistant bacteria. Thappeta et al. (2020) [99] reported that the CSM5K5 peptide works synergistically with traditional antibiotics against three antimicrobial-resistant pathogens. Furthermore, this peptide restored the susceptibility of MRSA USA300 to oxacillin, *E. faecalis* V583 to vancomycin, and MDR and ESBL-producing *E. coli* EC958 to streptomycin to concentrations equal to or below the clinical breakpoint for each antibiotic.

Finally, combination therapy with AMPs can reduce the risk of resistance development. Unlike conventional antibiotics, AMPs usually target multiple bacterial targets simultaneously, making it more difficult for bacteria to acquire stable resistance. Moreover, AMP–antibiotic combination therapy results in stronger antibacterial effects at lower doses and a lower likelihood of resistance [11,100].

These findings highlight that AMPs not only enhance antibiotic activity by direct antimicrobial effects but also suppress or prevent bacterial resistance mechanisms, making them promising candidates for combination therapies.

## 6. Evidence from In Vitro Studies

Many in vitro studies have highlighted the potential of combining AMPs with standard antibiotics. Through in vitro tests, it is possible to assess the activity of AMPs, their effects against various bacterial stages (biofilm or persister cultures), the minimum inhibitory concentration of the tested agent, cytotoxicity, and their activity in host tissue infection models. Additionally, these studies enable the identification of the mechanisms underlying possible synergy under controlled conditions as well as the action of AMP–antibiotic combinations [11,66]. Table 1 shows an example list of in vitro studies that demonstrated synergy, detailing the specific AMPs, antibiotics, organisms tested along with available quantitative measures of synergy. Although not all studies provide FICI values, the available data allow identification of general trends in AMP–antibiotic interactions.

The analyzed data demonstrated that AMP–antibiotic combinations exhibit broad synergistic activity against a wide range of bacterial species. Synergistic effects were observed for antibiotics with diverse mechanisms of action (e.g., β-lactams, aminoglycosides, fluoroquinolones) often in combination with multiple, structurally diverse AMPs. No combination was universally superior, highlighting the context-dependent nature of AMP–antibiotic synergy. Importantly, even subtle modifications in AMP structure could altered both the strength of synergy and the spectrum of antibiotics with which they interacted, highlighting the vast potential for designing new AMPs. The observed activity against multidrug-resistant and ESKAPE pathogens further emphasizes the potential of these combinations as promising strategies for future therapeutic applications.

The checkerboard assay is most often used to determine the presence of synergy between antimicrobial agents, which determines the fractional inhibitory index (FICI). This index is calculated as the sum of the FICs of agents A and B, where the FIC of agent A = (MIC of agent A in combination)/(MIC of agent A alone), and the FIC of agent B = (MIC of agent B in combination)/(MIC of agent B alone). Interactions between agents were classified as synergistic or antagonistic based on the FICI. Antagonism occurs when the inhibitory effect of the combined agents is weaker than that of the individual compounds. Synergy occurs when the inhibitory effect is greater than that of either compound alone. Synergy occurs when the value of FICI ≤ 0.5, no interaction is defined between agents when FICI = 0.5–4.0 and antagonism defined by FICI > 4.0 [34,92].

Another method often used to determine synergy is the time-kill assay. In this method, bacterial cultures are exposed to single agents and their combinations at defined concentrations, and viable counts (CFU/mL) are determined at specific time intervals to monitor bactericidal activity over time. Synergy is commonly defined as a ≥2-log_10_ reduction in colony count at 24 h for the combination compared to the most active single agent, whereas indifference is a change of less than 2-log_10_ and antagonism is a ≥2-log_10_ increase under combination conditions compared to the single most effective agent [32,110].

Although checkerboard and time-kill assays are the most commonly used methods to evaluate AMP–antibiotic interactions, both approaches present significant methodological challenges that deeply affect reproducibility and clinical translation. When comparing the two, the checkerboard assay is advantageous for its high-throughput screening capabilities; however, it is a static, endpoint-based method that fails to capture the dynamic interactions between agents over time. Conversely, the time-kill assay provides a dynamic assessment of bactericidal activity and is generally considered more sensitive, but it is labor-intensive and determining synergy can be complicated by the non-linear dose–response kinetics of the bacterial kill curves [33,106].

A critical insight often missing from current literature is the profound impact of methodological inconsistencies on the reported synergy. While checkerboard and time-kill assays are standard, they present significant challenges that hinder clinical translation. For instance, standard Mueller–Hinton broth contains variable concentrations of divalent cations that competitively inhibit cationic AMPs, drastically and artificially altering FICI values. Recognizing and standardizing these in vitro variables is a prerequisite before declaring true synergy or advancing to in vivo models.

A major issue with both assays is the high variability in reported synergy across different studies. This discrepancy frequently stems from a lack of methodological standardization. For instance, the choice of testing media critically influences AMP activity; standard Mueller–Hinton broth often contains variable concentrations of divalent cations (such as Mg^2+^ and Ca^2+^) that can competitively inhibit cationic AMPs from binding to the bacterial membrane, drastically altering the resulting FICI values. Additionally, variables such as starting inoculum size, incubation time, and the propensity of hydrophobic peptides to adhere to the polystyrene plastic of standard microtiter plates can artificially inflate minimum inhibitory concentrations (MICs) and mask true synergistic effects [111,112,113,114,115].

Ultimately, these methodological limitations severely impact the translation of in vitro findings to in vivo success. Standard laboratory conditions fail to replicate the complex physiological microenvironment of the host, which includes the presence of serum proteins, physiological salt concentrations, and varying pH levels—all of which can prematurely deactivate AMPs. Consequently, a synergistic interaction identified via a checkerboard assay in vitro may be completely neutralized in an in vivo setting, emphasizing the need to interpret FICI and time-kill data with caution and to test promising combinations in biologically relevant media prior to animal studies [33,107].

## 7. Evidence from In Vivo Studies

Even though AMPs are a promising alternative to conventional therapies, only a small number of strictly defined host defense peptides have been approved for clinical use by the FDA, with several still under clinical development [116,117]. However, if we broaden the scope to include microbial-derived peptide antibiotics and bacteriocins—which share the membrane-active properties of classical AMPs—agents such as gramicidin, polymyxins, nisin, and daptomycin are already successfully utilized in clinical practice [118]. While these are technically peptide antibiotics, their clinical success provides a strong proof-of-concept for the safety and viability of membrane-targeting peptide therapeutics [118,119].

A clear example of the successful translation of in vitro findings into in vivo efficacy was presented by Tao et al. (2024) [120], who investigated the antimicrobial peptide PMAP-36 in combination with antibiotics against porcine extraintestinal pathogenic *E. coli* (ExPEC). In vitro assays revealed that PMAP-36 acted synergistically with tetracycline, killing all bacteria within 30 min and leading to cell wall shrinkage, increased cell permeability, and bacterial cell death. The synergistic effect was then tested using a mouse model of infection. The combined treatment demonstrated a higher survival rate than antibiotics alone, reduced the bacterial load in the liver, spleen, lung, and blood, and protected the lungs from infection. Additionally, combined treatment with PMAP-36 and tetracycline promoted the recruitment of monocytes and macrophages to the abdominal cavity of mice.

Similarly, Otvos Jr. et al. (2018) [121] demonstrated both in vitro and in vivo synergistic effects of the proline-rich antimicrobial peptide A3-APO and colistin against multidrug-resistant *K. pneumoniae* and *A. baumannii*. The tested combination resulted in improved survival rates compared to monotherapy. Interestingly, in the in vivo model, the protective effect of A3-APO was not solely attributable to direct bacterial killing. Instead, the peptide exerted its therapeutic activity primarily through immunostimulatory mechanisms, enhancing the host’s innate immune response and indirectly promoting bacterial clearance.

In addition to their direct antimicrobial activity, AMPs may also exhibit anti-inflammatory and immunomodulatory activities by initiating adaptive immunity and stimulating immune cell chemotaxis and differentiation. They may also prevent inflammation by removing bacterial endotoxins and inhibiting cytokine release [122]. For example, human β-defensin 3 (hBD-3) has been shown to exert immunosuppressive activity both in vitro and in vivo, inhibiting T-cell activation and reducing the secretion of pro-inflammatory cytokines. In murine models, hBD-3 administration led to decreased inflammatory responses, confirming its dual role as both an antimicrobial and an immuno-modulatory peptide [123].

Moreover, some AMPs also promote wound healing by enhancing granulation tissue formation, collagen production, angiogenesis, and reepithelialization [116]. In a large animal model of diabetic wound healing, Hirsch et al. (2009) [124] characterized the effect of hBD-3 on diabetic wounds infected with *S. aureus*. They demonstrated that the use of Ad5-CMV-hBD-3 accelerated wound healing by 25% and resulted in a 10-fold reduction in bacterial growth at day 4. Synthetic IDR-1018 also have shown significant wound-healing potential. In a mouse model, IDR-1018 treatment accelerated wound closure and demonstrated significantly better wound reepithelialization than LL-37 or HB-107. The second study used a porcine wound healing model infected with *S. aureus*. IDR-1018 treatment significantly enhanced wound healing/reepithelialization and keratinocyte migration, in the absence of direct antimicrobial activity [125].

From a safety perspective, in vivo studies have indicated that many AMPs are well-tolerated at therapeutic doses, although toxicity and stability remain major limitations for systemic use. To enhance the safety and stability of AMPs, ongoing research focuses on chemical modifications, the design of synthetic peptide analogs, and the development of nanoparticle-based delivery systems [119].

These findings highlight that the potentiating effects of AMPs observed under controlled in vitro conditions can successfully translate into therapeutic benefits in living organisms. However, it is important to remember that despite promising preclinical outcomes, the successful translation of AMPs into clinical practice remains limited. Such challenges are not unique to peptide-based therapeutics, as many drug candidates that perform well in controlled experimental settings ultimately fail to demonstrate comparable efficacy or safety in clinical trials. This persistent gap between preclinical promise and clinical applicability is well-illustrated by several high-profile clinical trials. For instance, murepavadin (POL7080), a highly specific synthetic peptide targeting the LptD protein in Gram-negative bacteria, progressed to Phase III clinical trials for the treatment of *Pseudomonas aeruginosa* infections. However, the trial was prematurely discontinued due to unacceptably high rates of renal toxicity, highlighting how systemic nephrotoxicity remains a primary barrier for parenterally administered peptides [126]. Similarly, pexiganan, a synthetic analogue of magainin intended for topical application, reached Phase III trials for the treatment of infected diabetic foot ulcers. While it demonstrated a favorable safety profile, it ultimately failed to secure FDA approval because it did not demonstrate superiority over standard, less expensive systemic antibiotic therapies (e.g., oral fluoroquinolones) [127]. Conversely, omiganan (an indolicidin synthetic analogue) successfully completed Phase II trials for atopic dermatitis, largely because its topical application localized the therapeutic effect, entirely bypassing systemic pharmacokinetic degradation and toxicity [128]. These mixed clinical outcomes underscore a critical lesson for combination therapies: demonstrating in vitro synergy is insufficient. Future clinical success will heavily depend on selecting combinations where the AMP component is restricted to a localized site or engineered to withstand systemic clearance without inducing host cytotoxicity.

## 8. Challenges and Limitations

Despite the promising results of AMP–antibiotic combination therapies, several challenges hinder their translation from laboratory studies to clinical practice. The effectiveness of antibiotic-AMP combinations and the synergy between them depends on many factors. One of the main issues is the variability in observed synergy, which depends strongly on the type of AMP, antibiotic used, and bacterial strain tested. A combination that displays strong synergy against one pathogen may show indifference or even antagonism against another. Furthermore, although most AMPs demonstrate strong in vitro activity, these results may not translate into antibacterial activity in vivo due to the complexity of the microenvironment [11,47].

Three other major problems with the use of AMPs are their stability, toxicity, and production costs [89]. AMPs are characterized by a short half-life in the clinical environment—both in the gastrointestinal tract and blood—as they are susceptible to degradation by proteolytic enzymes. A primary barrier to the systemic administration of AMPs lies in their highly unfavorable pharmacokinetic (PK) profiles. Due to their peptide nature, AMPs typically exhibit a very short plasma half-life—often in the range of mere minutes—which severely limits their systemic bioavailability. This rapid clearance is driven by ubiquitous proteolytic enzymes (e.g., trypsin, pepsin, and serum proteases) that readily cleave the peptide bonds. Furthermore, high affinity for plasma proteins (such as albumin) can sequester AMPs, significantly reducing the fraction of unbound, active peptide available to reach the infection site. Even if a peptide survives proteolytic degradation, its cationic nature often leads to rapid renal clearance and hepatic sequestration [24,129,130]. Moreover, AMPs may lose their bactericidal activity under physiological salt conditions, due to the loss of electrostatic interactions with the cell membranes [118,122]. For this reason, most currently used AMPs are intended for topical use, especially on wounds [116].

Another important limitation of AMPs is their toxicity. Potential toxicity depends on many factors, including concentration, route of administration, target cells, peptide sequence, and mechanism of action. AMPs can exert toxicity by acting on membranes or binding to receptors [23]. Peptides that act on membranes are not entirely selective towards microorganisms and can be toxic to eukaryotic cells [118]. However eukaryotic cell membranes are mostly composed of zwitterionic phospholipids, with anionic phospholipids mainly confined to the inner leaflet of the bilayer. This asymmetric organization significantly reduces the strength of electrostatic interactions between cationic AMPs and host cells. Additionally, the presence of cholesterol in mammalian membranes and the fact that bacterial membrane potential is more negative further decrease AMP interactions with mammalian cells, making such interactions generally weak under physiological conditions [131,132]. Many AMPs display potent antimicrobial activity at nanomolar to low micromolar concentrations [44,133,134], but the optimal therapeutic concentration remains difficult to define because of substantial variability between peptides and target pathogens. However, when these protective features are overcome, such as at higher peptide concentrations or in particularly susceptible cell types, cationic AMPs can interact with host membranes and induce cytotoxic effects. Cationic AMPs can induce hemolysis by interacting with negatively charged ions on the surface of erythrocytes, leading to the formation of oligomers and cell destruction. Furthermore, there are known examples of AMPs that are nephrotoxic in vivo following parenteral administration. Antimicrobial peptides can be toxic to renal tubules by inducing oxidative stress, apoptosis, cell cycle arrest and autophagy [119]. In addition, high concentrations of AMPs that bind to receptors can dysregulate the immune system, leading to excessive immune responses and triggering inflammatory secondary diseases such as rosacea, psoriasis or atopic dermatitis [23].

Therefore, the routes of administration of AMP-based agents are challenging and remain a major limitation for their clinical translation. Most antimicrobial peptide-based drugs are currently limited to topical or intravenous administration [135]. Due to their limited stability and bioavailability, oral administration is largely ineffective, as AMPs are poorly stable at gastric pH and susceptible to proteolytic enzymes [117,136]. To better utilize AMPs as therapeutic agents, further research is required on delivery systems that will not only increase bioavailability but also reduce cytotoxicity and increase efficacy through increased specificity and solubility [118].

The development and use of AMPs as pharmaceuticals is also limited by high production costs resulting from the high price of raw materials, complex preparation processes, which usually involve the chemical synthesis of peptides, and relatively low process efficiency. Although chemical synthesis ensures high purity and control over the entire production process, it is expensive, especially for the production of longer peptide chains. The high production price translates into a high product price, which means that drugs based on AMPs may be uncompetitive in the market, especially compared to antibiotics that are cheap to produce on a large scale [135]. In nature, antimicrobial peptides rarely act as isolated agents. Most organisms produce many different AMPs that cooperate synergistically to fight infections [137,138]. Synergistic AMP combinations may also help reduce production costs, as lower doses of each peptide are required to achieve therapeutic efficacy [31]. Such a nature-inspired formula may represent a promising opportunity to improve the availability and cost-effectiveness of AMP-based drug production.

Excessive use of antibiotics has led to the development of bacterial resistance, and although this risk is considerably lower with AMPs due to their multi-target and rapid modes of action, it cannot be completely excluded. Bacteria can evolve resistance to AMPs through several adaptive mechanisms, such as modifying their membrane or cell wall to reduce surface charge, producing protective capsules, secreting proteolytic enzymes, or employing efflux pumps to expel peptides [134]. For this reason, increasing attention is being focused on multi-target peptide strategies, including combinations of AMPs that simultaneously compromise membrane integrity and disrupt intracellular processes such as transcription and translation. By acting on multiple essential bacterial pathways at once, peptide complexes further reduce the likelihood of resistance and enhance antibacterial efficacy [11,88,139].

Overall, while AMPs represent a promising avenue for overcoming antibiotic resistance, addressing these challenges—variability in activity, limited stability and delivery and the potential for adaptive resistance—will be critical for their successful clinical implementation.

## 9. Future Perspectives and Applications

Due to the potential use of AMPs, recent research has focused on improving their properties through structural and chemical modifications and innovative delivery strategies. One of the most common approaches is amino acid substitution, in which natural L-amino acids are replaced with their D-enantiomers to increase proteolytic resistance [140,141]. Another widely used method is cyclization, which involves the formation of cyclic structures using disulfide and/or amide bonds, or lactamization, which makes peptides less susceptible to proteases, more stable, and exhibits greater antimicrobial activity and selectivity [142]. In turn, C-terminal amidation and stapling, which involves the formation of a rigid α-helix conformation by cross-linking the side chains, increase the antibacterial activity and stability of the peptides. In addition to increasing stability, N-terminal acetylation of antimicrobial peptides provides better resistance to enzymatic degradation [140].

Other modifications to antimicrobial peptides involve the addition of various external chemical groups. Glycation involves covalently linking polysaccharides to peptides, making them less susceptible to proteases, facilitating their folding, and improving their amphiphilicity [135]. PEGylation, or the attachment of a polyethylene glycol polymer, enhances solubility and stability, increases plasma half-life, protects against proteolysis, and reduces toxicity and immunogenicity [143]. Similarly, lipidation, involving the attachment of fatty acid chains, can increase and modulate hydrophobicity, enzymatic stability, bioavailability, and bioactivity [144].

In addition to chemical optimization, the development of advanced delivery systems—such as nanoparticles, liposomes, hydrogels, and polymeric matrices—represents a promising direction for clinical translation of these compounds. The use of nanoparticles conjugated with antimicrobial peptides can mitigate peptide toxicity while simultaneously enhancing stability and bioavailability. Certain metal nanoparticles possess inherent antimicrobial properties that can further strengthen the therapeutic potential of AMP-based systems. Among them, gold nanoparticles (AuNPs) have attracted particular attention due to their excellent biocompatibility and low toxicity, while silver nanoparticles (AgNPs) are well known for their potent antibacterial activity. Moreover, as nonbiological entities, these nanoparticles are not readily recognized by the immune system, which further increases their suitability for therapeutic applications [142,145].

To bridge the gap between in vitro synergy and clinical viability, advanced delivery systems must be leveraged to directly counter the pharmacokinetic and toxicity barriers of AMPs. Nanoparticle-based delivery systems, liposomes, and polymeric matrices are no longer just optional enhancements; they are prerequisites for systemic AMP therapy. For example, encapsulating AMPs within liposomes or poly(lactic-co-glycolic acid) (PLGA) nanoparticles shields the peptides from premature proteolytic degradation in the bloodstream, significantly extending their circulating half-life [111]. Furthermore, these nanocarriers can be engineered to exploit the enhanced permeability and retention (EPR) effect at sites of infection and inflammation, providing targeted delivery. This localization is crucial because it allows for a high local concentration of both the AMP and the synergistic antibiotic at the infection site, while maintaining low systemic circulating levels—thereby circumventing the nephrotoxic and hemolytic side effects that have derailed previous clinical trials. Additionally, modern delivery platforms allow for the co-encapsulation of both the AMP and the conventional antibiotic within the same carrier. This ensures that both agents reach the target bacteria simultaneously and in the exact synergistic molar ratios optimized during in vitro checkerboard assays, maximizing their combined therapeutic potential [146,147,148].

Another promising polymeric carrier is poly(γ-glutamic acid) (PGA), a naturally derived, biodegradable polypeptide capable of forming stable nanoparticles that can enhance peptide stability and controlled release, making it a useful platform for AMP delivery [149]. Liposomes are spherical nanocarriers consisting of one or more lipid bilayers that can encapsulate both hydrophilic and hydrophobic AMPs. Their biocompatible lipid composition and structural versatility make them an attractive system for AMPs delivery, as they can protect peptides from enzymatic degradation and allow for controlled release. By modifying lipid composition, size, or surface charge, liposomes can be optimized to improve AMPs stability, bioavailability, and selective targeting [145].

An interesting strategy is the use of hydrogels, which are particularly useful as dressings for damaged skin treatment. Hydrogels are three-dimensional polymeric networks capable of retaining large amounts of water, giving them structural and mechanical properties similar to natural tissues. Their hydrated matrix provides a favorable microenvironment for antimicrobial peptides, protecting them from enzymatic degradation and denaturation. These systems enable sustained peptide release, enhance local bioavailability, and reduce cytotoxicity, making hydrogels promising carriers for topical and wound-healing applications [117,150].

Considering their structural and chemical versatility, the modifications and delivery strategies described above represent a key approach to overcoming the major limitations associated with the therapeutic use of AMPs, such as instability, cytotoxicity, and low bioavailability. These advances may not only improve the pharmacological properties of AMPs but also enable better utilization in antimicrobial therapies [118].

Given the growing threat of bacterial resistance to conventional antibiotics, AMP-based therapies hold significant promise as synergistic agents in combination therapy. Such strategies often enhance the efficacy of antibiotics, allowing the use of lower drug doses compared to monotherapy, which can minimize adverse effects and reduce the selective pressure that drive resistance. In addition, combination approaches can extend the antimicrobial spectrum, improve patient safety, and even allow the reuse of older antibiotics that had been abandoned because of their toxicity or ineffectiveness [22].

Beyond their antimicrobial role, AMPs are increasingly recognized for their immunomodulatory potential. They play a role in regulating the immune responses and inflammatory processes by modulating signaling pathways, activating immune cells and promoting their proliferation, enhancing cytokine and immunoglobulin secretion, and promoting processes such as autophagy, apoptosis, and wound healing [151]. These properties suggest that AMPs could serve not only as antimicrobial agents but also as valuable adjuncts in immunotherapies—supporting host defense, reducing inflammation, and promoting tissue regeneration [152]. In the future, AMPs may become part of integrated therapeutic strategies combining antimicrobial, anti-inflammatory and immunomodulatory functions.

## 10. Conclusions

The growing threat of bacterial resistance to conventional antibiotics has led to a need to search for new therapeutic agents. The lack of new antibiotics has shifted research interest towards alternative solutions. Antimicrobial peptides (AMPs) are gaining increasing attention because of their diverse mechanisms of action, broad spectrum of activity, low probability of inducing resistance, and immunomodulatory properties. A particularly significant advantage of using AMPs in treatment is their potential for combination therapy with conventional antibiotics. By combining the membrane-disrupting or intracellular targeting activities of AMPs with traditional antibiotic mechanisms, these therapies can enhance antimicrobial effectiveness while simultaneously reducing the required doses.

Several in vitro and in vivo studies have demonstrated that AMP–antibiotic combinations can act synergistically, thereby improving treatment efficacy against resistant strains. This synergy has been observed through various mechanisms, including increased membrane permeability, interference with intercellular processes and biofilm formation, and efflux pump inhibitions. In addition, AMPs can modulate host immune responses, reduce inflammation, and promote tissue repair, providing multifaceted benefits that extend beyond direct antimicrobial activity.

Despite these promising results in research, several challenges must be addressed before AMP-based combination therapies can be widely applied in clinical practice. It is particularly important to develop strategies to solve problems related to peptide stability, cytotoxicity, delivery systems, efficient production and the standardization of synergy testing methods. Solving these problems involves research on peptide optimization and modifications, the development of innovative delivery routes, and further in vivo evaluation of safety and efficacy. Nevertheless, the results already indicate that AMPs, particularly in synergistic combinations with antibiotics, may represent a promising frontier in the fight against multidrug-resistant bacterial infections.

## Figures and Tables

**Table 1:** In vitro studies of AMPs in combination with antibiotics.

AMPs	Antibiotic	Organism	FICI	Reference
Tachyplesin III	imipenem	*P. aeruginosa* ATCC 27853	0.312	[101]
LL-37	amoxicillin with clavulanic acid	*S. aureus* (5 strains)	N/A	[78]
Pleurocidin	ampicillin	*S. aureus* ATCC 25923	0.5	[94]
		*P. acnes* ATCC 6919	0.375	
		*P. aeruginosa* ATCC 27853	0.375	
		*E. coli* ATCC 25922	0.5	
		*E. coli* O-157 ATCC 43895	0.375	
	erythromycin	*S. aureus* ATCC 25923	0.375	
		*P. acnes* ATCC 6919	0.375	
		*E. faecium* ATCC 19434	0.5	
		*P. aeruginosa* ATCC 27853	0.375	
		*E. coli* ATCC 25922	0.375	
		*E. coli* O-157 ATCC 43895	0.5	
	chloramphenicol	*S. aureus* ATCC 25923	0.5	
		*P. acnes* ATCC 6919	0.375	
		*E. faecium* ATCC 19434	0.5	
		*P. aeruginosa* ATCC 27853	0.375	
		*E. coli* ATCC 25922	0.5	
		*E. coli* O-157 ATCC 43895	0.5	
Arenicin-1	ampicillin	*S. aureus* ATCC 25923	0.5	[102]
		*S. epidermidis* KCTC 1917	0.5	
		*P. aeruginosa* ATCC 27853	0.5	
		*E. coli* ATCC 25922	0.375	
		*E. coli* O-157 ATCC 43895	0.5	
	erythromycin	*S. aureus* ATCC 25923	0.375	
		*S. epidermidis* KCTC 1917	0.5	
		*E. faecium* ATCC 19434	0.5	
		*P. aeruginosa* ATCC 27853	0.5	
		*E. coli* ATCC 25922	0.375	
		*E. coli* O-157 ATCC 43895	0.5	
	chloramphenicol	*S. aureus* ATCC 25923	0.5	
		*S. epidermidis* KCTC 1917	0.375	
		*E. faecium* ATCC 19434	0.375	
		*P. aeruginosa* ATCC 27853	0.375	
		*E. coli* ATCC 25922	0.5	
		*E. coli* O-157 ATCC 43895	0.375	
Nisin Z	penicillin G	*P. fluorescens* LRC-R73	≤0.01	[103]
	streptomycin		≤0.01	
	lincomycin		≤0.01	
	vancomycin		≤0.01	
	lysozyme		0.02	
	kanamycin		0.25	
	rifampicin		0.07	
	chloramphenicol		≤0.01	
Pediocin PA-1/AcH	penicillin G		≤0.01	
	streptomycin		≤0.01	
	lincomycin		0.02	
	vancomycin		≤0.01	
	lysozyme		0.02	
	kanamycin		0.2	
	rifampicin		0.07	
	chloramphenicol		0.03	
C12(ω7)K-β12	tetracycline, erythromycin	*E. coli* AG100	N/A	[98]
NZ2114	amoxicillin, penicillin, flucloxacillin, gentamicin, neomycin, amikacin	methicillin-sensitive *S. aureus* (MSSA)	N/A	[97]
		methicillin-resistant *S. aureus* (MRSA)		
CLP-19	ampicillin	*E. coli* ATCC 25922	0.375	[95]
		*S. aureus* ATCC 29213	0.5	
	ceftazidime	*E. coli* ATCC 25922	0.5	
		*S. aureus* ATCC 29213	0.5	
		*A. baumannii* ATCC 19606	0.5	
	levofloxacin	*S. aureus* ATCC 29213	0.5	
		*E. coli* ATCC 25922	0.5	
FK-13-a1 FK-13-a7	chloramphenicol	methicillin-resistant *S. aureus* CCARM 3095	N/A	[104]
		*P. aeruginosa* CCARM 2095		
AamAP1-Lysine	rifampicin	*S. aureus* ATCC 29213	0.203	[105]
		*S. aureus* ATCC 33591	0.123	
	erythromycin	*S. aureus* ATCC 29213	0.204	
	levofloxacin	*S. aureus* ATCC 33591	0.103	
		*P. aeruginosa* ATCC BAA2114	0.36	
	chloramphenicol	*P. aeruginosa* ATCC BAA2114	0.36	
Melimine	ciprofloxacin	ciprofloxacin resistant *P. aeruginosa* 37	0.31–0.38	[106]
Protegrin-1	colistin	*A. baumannii* (21 strains)	N/A	[107]
	fosfomycin	*A. baumannii* (9 strains)		
	meropenem	*A. baumannii* (9 strains)		
	tigecycline	*A. baumannii* (5 strains)		
Indolicidin	polymyxin B	MDR *P. aeruginosa* PA 910	0.5	[92]
	tobramycin		0.5	
	gentamycin		0.5	
	amikacin		0.5	
	tetracyclin		0.5	
Indopt 3	polymyxin B		0.38	
	meropenem		0.38	
	tobramycin		0.38	
	gentamycin		0.25	
	amikacin		0.31	
L11W	penicillin	MRSE *S. epidermidis* 1208	0.3121	[108]
	ampicillin		0.2808	
	erythromycin		0.2808	
L12W	penicillin		0.2808	
	ampicillin		0.2574	
	erythromycin		0.2808	
I4WL5W	penicillin		0.1875	
	ampicillin		0.1562	
	erythromycin		0.3124	
I1WL5W	penicillin		0.2812	
	ampicillin		0.2578	
	erythromycin		0.2812	
	tetracycline		0.2820	
L12	vancomycin	MRSA *S. aureus* S26	0.375	[109]
	levofloxacin		0.313	
	vancomycin	MRSA *S. aureus* S49	0.188	
	levofloxacin		0.125	
Ano-1β	rifampicin	*P. aeruginosa* ATCC 27853	0.3125	[76]
	polymyxin B		0.3125	
Ano-8β	rifampicin		0.3125	
LL-37	polymyxin B	*E. coli* MG1655	0.37	[87]
		*P. aeruginosa* PAO1	0.31	

N/A—not applicable; FICI was not reported and could not be calculated. Some studies used alternative indices with different interpretation criteria.

## Data Availability

No new data were created or analyzed during this study. Data sharing is not applicable to this article.
